# The Potential of *Mangifera indica* L. Peel Extract to Be Revalued in Cosmetic Applications

**DOI:** 10.3390/antiox12101892

**Published:** 2023-10-21

**Authors:** Abigail García-Villegas, Álvaro Fernández-Ochoa, Alejandro Rojas-García, María Elena Alañón, David Arráez-Román, María de la Luz Cádiz-Gurrea, Antonio Segura-Carretero

**Affiliations:** 1 Department of Analytical Chemistry, University of Granada, Av. Fuentenueva s/n, 18071 Granada, Spain; abigarcia@ugr.es (A.G.-V.); alvaroferochoa@ugr.es (Á.F.-O.); alejorogar@ugr.es (A.R.-G.); darraez@ugr.es (D.A.-R.); ansegura@ugr.es (A.S.-C.); 2 Regional Institute for Applied Scientific Research (IRICA), University of Castilla-La Mancha, Avda. Camilo José Cela 10, 13071 Ciudad Real, Spain; mariaelena.alanon@uclm.es; 3 Department of Analytical Chemistry and Food Science and Technology, University of Castilla-La Mancha, Ronda de Calatrava 7, 13071 Ciudad Real, Spain

**Keywords:** mango, by-products, phenolic compounds, antioxidants, skin health, anti-aging, cosmeceuticals

## Abstract

The constant growth of the cosmetic industry, together with the scientific evidence of the beneficial properties of phytochemicals, has generated great interest in the incorporation of bioactive extracts in cosmetic formulations. This study aims to evaluate the bioactive potential of a mango peel extract for its incorporation into cosmetic formulations. For this purpose, several assays were conducted: phytochemical characterization; total phenolic content (TPC) and antioxidant potential; free-radical scavenging capacity; and skin aging-related enzyme inhibition. In addition, the extract was incorporated into a gel formulation, and a preliminary stability study was conducted where the accelerated (temperature ramp, centrifugation, and heating/cooling cycles) and long-term (storage in light and dark for three months) stability of the mango peel formulations were evaluated. The characterization results showed the annotation of 71 compounds, gallotannins being the most representative group. In addition, the mango peel extract was shown to be effective against the ^•^NO radical with an IC_50_ of 7.5 mg/L and against the hyaluronidase and xanthine oxidase enzymes with IC_50_ of 27 mg/L and 2 mg/L, respectively. The formulations incorporating the extract were stable during the stability study. The results demonstrate that mango peel extract can be a by-product to be revalorized as a promising cosmetic ingredient.

## 1. Introduction

Aging manifests itself progressively over the years and is characterized by a reduction in the quality of life of the organism. In fact, it is the main risk factor for the development of many disorders, including cardiovascular, neurodegenerative, and skin diseases [1]. In this context, aging is manifested noticeably in the skin due to changes in its structure, function, and appearance. Skin aging remains a rather complex and poorly understood process [2]. Unlike other organs, the skin is not only affected by the intrinsic aging process that occurs in all tissues but also by extrinsic environmental factors that accelerate the aging process [3,4].

Intrinsic aging is regulated by genetic factors and is characterized by thinning of the epidermis and dermis, a decrease in the production of keratinocytes and fibroblasts, dryness, the appearance of fine wrinkles, and increased sensitivity and fragility. On the other hand, extrinsic aging is mainly caused by environmental factors, such as ultraviolet (UV) light, which generate reactive oxygen species (ROS) that play an important role in skin aging and whose accumulation induces oxidative stress and can cause loss of lipids and proteins and DNA and organelle damage, leading to cellular senescence, the main mechanism involved in skin aging [5]. In addition, UV can also cause abnormal pigmentation and sunburn [3]. Generally, skin aging leads to loss of physiological function and decreased structural integrity, increasing the risk of skin diseases [3,6].

In recent years, there has been a notable interest among the population in caring for and maintaining healthy skin due to the passing of the years and the harmful effects that environmental factors can cause [7]. Concern for skin care has led to a demand for natural and safe products that guarantee real efficacy for skin health [8]. Plants are abundant sources of phenolic compounds and natural pigments, which makes them very interesting for cosmetic applications [9].

Recently, bioactive compounds such as phenolic compounds have been reported to exhibit antioxidant, anti-inflammatory, and anti-aging activity on the skin due to their ability to scavenge or reduce ROS levels and provide UV photoprotection [10]. Phenolic compounds have been used as sunscreen ingredients due to their anti-UV capacity [11]. The UV absorption profile of these compounds will depend on the presence and number of aromatic rings, double bonds, stereochemistry, and type of substituents [12]. For example, soluble and insoluble polyphenols are able to absorb radiation in the range of 304–350 and 352–385 nm, respectively [12]. Therefore, the incorporation of some of these compounds in sunscreens will increase the SPF value and improve photostability [11,12]. In addition, phenolic compounds such as flavonoids have been shown to reduce collagen degradation, enhance the healing process, mitigate skin hyperpigmentation, and intervene in signaling pathways associated with inflammatory reactions [10].

Therefore, the use of natural extracts rich in polyphenols is being investigated for the development of skin products [13]. Moreover, environmental awareness and the reduced use of synthetic products have led the cosmetic industry to develop new natural cosmetic products with positive effects on the skin to prevent possible skin damage and pathologies [14].

Mango (*Mangifera indica* L.) is a tropical fruit belonging to the *Anacardiaceae* family appreciated for its excellent organoleptic characteristics and nutritional properties. This product represents one of the most important tropical fruits grown and distributed worldwide with more than 26 million tons per year [15]. Mango is not only consumed fresh but also marketed in different formats according to consumer needs. Because of its processing for the commercialization of different mango products, large amounts of waste, mainly peels and seeds, are generated and discarded [16]. In this sense, mango peel represents approximately 15–20% of the total fruit [16,17]. Considering that approximately 20% of the world’s mango production is used to produce different products and that mango peel accounts for 15–20% of the total fruit, approximately 1 million tons of mango peel will be generated worldwide per year [18]. However, it is worth noting that mango peel may be an interesting source of phenolic compounds, even in higher proportion than the pulp. The main bioactive compounds found in mango peel are polyphenols, carotenoids, and organic acids [16]. These compounds exhibit potent antioxidant, anti-inflammatory, and anti-aging activity [19]. Moreover, several scientific investigations have reported on the functional properties and therapeutic uses of mango by-products [20,21,22]. Therefore, mango peel could be a by-product from which to obtain a wide variety of ingredients with functional potential for the formulation of cosmetic products.

Based on this context, this study focused on evaluating mango peel as a possible phytochemical source and its revalorization for obtaining bioactive ingredients for the formulation of cosmeceuticals. To this end, the phenolic composition of mango peel extract was characterized, and an in-depth in vitro study was carried out to evaluate its total phenolic content, its antioxidant activity, and its ability to inhibit ROS/RNS and enzymes. In addition, the effect of mango peel extract on pigmentation was also evaluated, and the formulation of a cosmetic prototype incorporating mango peel extract was carried out and its stability studied.

## 2. Materials and Methods

### 2.1. Chemical and Reagents

All chemicals were of HPLC-MS grade and used as received. Formic acid, water, and acetonitrile for HPLC platforms were purchased from Fluka (Sigma-Aldrich, Steinheim, Germany) and Lab-Scan (Gliwice, Sowinskiego, Poland), respectively. Milli-Q Milli-pore (Bedford, MA, USA) ultrapure water and absolute ethanol were purchased from VWR Chemicals (Radnor, PA, USA) and used for extractions and solutions.

To measure the TPC and antioxidant capacity, the following reagents were provided from the indicated suppliers: Folin–Ciocalteu reagent, gallic acid, AAPH (2,2’-azobis(2-amidinopropane), ABTS (2,2′-azinobis(3-ethylbenzothiazoline-6-sulfonate)), Trolox (6-hydroxy-2,5,7,8-tetramethylchroman-2-carboxylic acid) ferric chloride, ferrous sulphate heptahydrate, fluorescein, sodium phosphate monobasic and dibasic DHR (dihydrorhodamine), DMF (dimethylformamide), potassium dihydrogen phosphate anhydrous, NADH (β-nicotinamide adenine dinucleotide), NBT (nitrotetrazolium blue chloride), DAF-2 (diaminofluorescein diacetate) tyrosinase inhibitor screening kit (colorimetric), neutrophil elastase colorimetric drug discovery kit, Cayman’s xanthine oxidase fluorometric assay kit, sodium chloride, hyaluronic acid, hyaluronidase from sheep testes, tricine, 1–10 phenantroline, collagenase from Clostridium histolyticum, FALGPA (N-[3-(2-furyl)acryloyl]-L-leucyl-glycyl-L-prolyl-L-alanine), thrombin receptor activating peptide 6 (TRAP-6), sodium citrate 3.2%, and phosphate buffer solution (PBS) were purchased from Sigma-Aldrich (St. Louis, MO, USA). Collagen was purchased from Chrono-Log Corp. (Havertown, PA, USA). NOC-5 was obtained from Chemcruz (Santa Cruz Biotech., Dallas, TX, USA). Glycerine, xanthan gum, and citric acid were obtained from Guinama S.L.U (Valencia, Spain).

### 2.2. Mango Peel Extraction Process

Ripe mango fruits were donated by the company Grupo La Caña, Miguel García Sánchez e Hijos, SA (Granada, Spain). The mango peels were manually separated from the pulp and cleaned with water to remove any residue. Once cleaned, the peels were chopped, weighed, and dried for 9 h at 80 °C in a UF110 oven (Memmert GmbH + Co. KG, Schwabach, Germany). After drying, the shells were ground and sieved with a ZM 200 mill (Retsch GmbH, Haan, Germany). A fine, homogeneous powder with a particle size of less than 1 mm was obtained in order to facilitate the extraction process.

The extraction process was carried out by solid-liquid extraction. In dark glass vials, 10 g of the powdered by-product was weighed and 100 mL of ethanol and water in 80:20 (*v*:*v*) was added. Then, the glass vials were placed for 2 h on a magnetic stirrer at 45 °C and 170 rpm for proper mixing. All supernatants obtained were collected, filtered, and evaporated to dryness using a Savant SC250EXP SpeedVac concentrator (Thermo Scientific, Waltham, MA, USA). The dried extracts were weighed and stored at −20 °C in the dark until further use.

The conditions used to obtain the extract were appropriate and had no negative impact on the target compounds. The extraction process was based on previous reports but incorporated some modifications [23].

### 2.3. HPLC-ESI-qTOF-MS Analysis

To determine the chemical profile of the mango peel extract, HPLC-ESI-qTOF-MS analysis was performed. For this, the mango peel extract was reconstituted to a concentration of 5 mg/mL. The solution was then centrifuged, filtered (0.2 µm), and transferred to an HPLC vial for analysis. In addition, blank samples were prepared for contaminant detection.

The instrument used was an HPLC 1290 system coupled to a quadrupole time-of-flight (QTOF) mass spectrometer (G6530C UHD, Agilent Tech., Santa Clara, CA, USA) equipped with a Jet Stream dual ESI interface. Chromatographic separation was performed on an analytical C18 column (ACQUITY UPLC BEH Shield RP18 Column, 130 Å, 1.7 µm, 2.1 mm × 150 mm). The mobile phases were acidified water with 0.1% *v*/*v* formic acid (A) and acetonitrile (B). The following linear elution gradient was used: 0 min, 100% A; 5 min, 90% A; 18 min 15% A; 24 min, 0% A; 25.50 min, 0% A; 26.50 min, 95% A; 32.50 min, 95% A. The flow rate and the injection volume were 0.4 mL/min and 5 μL, respectively. The MS acquisition was performed in negative ionization mode and full scan mode covering a mass-to-charge ratio from 50 to 1200 *m*/*z*. In addition, the samples were also analyzed in a DDA MS/MS acquisition mode using fixed collision energies of 10.00, 30.00, and 60.00 eV. The source parameters were optimized as follows: gas temperature 200 °C, scan duration 1.2 s, desolvation gas flow resolution 10 L/min, nebulizer 20 psig, desolvation temperature 350 °C, capillary voltage 4 kV, nozzle voltage 500 V, and nebulization gas pressure 2 bar.

### 2.4. HPLC-ESI-qTOF-MS Data Processing

The acquired raw data were firstly transformed using the MSConverGUI software (https://proteowizard.sourceforge.io/download.html) and then processed in MZmine 2.53 to carry out different data processing steps such as background noise detection, ADAP chromatogram builder, ADAP chromatogram deconvolution, isotope grouping, and alignment. The following parameters were used to construct the ADAP chromatograms; intensity threshold 1.0 × 10^3^; highest minimum intensity 1.0 × 10^4^; *m*/*z* tolerance 25 ppm. For the deconvolution of the chromatogram, the wavelets algorithm (ADAP) and the following parameters were used: S/N threshold 10; minimum peak height 1.0 × 10^4^; area threshold 20; peak duration range 0.00–10.00; RT wavelet width range 0.00–15.00; *m*/*z* range for MS2 scan matching 25 ppm; RT range for MS^2^ scan matching 0.20 min. For isotopic peak clustering: *m*/*z* tolerance 25 ppm; RT tolerance 0.1 min; monotonic shape and maximum charge 2 were used. Finally, the chromatograms were aligned using the “Join Aligner” algorithm with an *m*/*z* tolerance of 25 ppm and an RT tolerance of 0.15 min. The program Sirius 5.8.1 was used to predict molecular formulas and chemical structures. The information obtained from Sirius was contrasted with the literature to annotate the compounds. Based on the identification guidelines suggested by Sumner et al., the compounds were categorized as follows: level 1 annotation was undertaken using commercial standards, level 2 annotation involved comparing the MS/MS spectra with those available in the databases, and level 3 annotation was based on the molecular formulation and MS1 spectra. Those signals that could not be annotated were reported as unknowns (level 4) [24].

### 2.5. Evaluation of the Mango Peel Extract Potential

Bioactivity assays were carried out with a Synergy H1 multimode microplate reader with monochromator-based optics (Bio-Tek Instruments Inc., Winooski, VT, USA). Ninety-six well microplates were used for the assays.

#### 2.5.1. Evaluation of Total Phenolic Content and Antioxidant Capacity

The TPC of mango peel extract was determined by the Folin–Ciocalteu spectrophotometric method described by Cádiz-Gurrea et al. [25]. The results were expressed as milligrams of gallic acid per gram of dry extract (DE). The antioxidant activity of the obtained mango peel extract was evaluated by ferric reducing antioxidant power (FRAP), trolox equivalent antioxidant capacity (TEAC), and oxygen radical absorbance capacity (ORAC) assays. The methods were carried out following previously described procedures with slight modifications [26,27,28]. The FRAP method was used for the determination of the antioxidant power through the reduction of the ferric cation to ferrous. The results were expressed as mmol of FeSO_4_ equivalents per gram of DE. The TEAC assay is based on the reduction of the ABTS* + radical by mango peel extract at different concentrations. Trolox was used as a standard. The results were expressed as µmol equivalents of Trolox per gram of DE. The ORAC method was performed according to the procedure described by Huang et al. in which the ability of the extract to scavenge the peroxyl radical is evaluated [27]. ORAC values were obtained by a regression equation between fluorescence decrease and Trolox concentration. Results were expressed as mmol of Trolox equivalents per gram of DE. All measurements were carried out in triplicate.

#### 2.5.2. Assessment of ROS/RNS Scavenging Activity

The ROS/RNS assays were performed in a 96-well microplate. The methodology previously described by Gomes et al. and Pinto et al. was used [29,30].

To measure the scavenging capacity of the superoxide radical, the radical was generated by the NADH/PMS and O_2_ system. The scavenging activity was determined colorimetrically at 560 nm on the plate reader by the reduction of NBT to diformazan purple. The results were expressed as the inhibition, in IC_50_, of the reduction of NBT to diformazan.

^•^NO scavenging capacity was performed by NO oxidation of non-fluorescent DAF-2 to fluorescent triazolofluorescein (DAF-2T). In the plate reader at 37 °C, fluorescence measurement was performed at 528 ± 20 nm with excitation at 485 ± 20 nm. The results were expressed as IC_50_ of NO-induced DAF-2 oxidation inhibition. To determine the scavenging ability of hypochlorous acid (HOCl), HOCl induced the oxidation of DHR to fluorescent rhodamine 123. It was measured in the microplate reader at a temperature of 37 °C and at an emission wavelength of 528 ± 20 nm with excitation at 485 ± 20 nm. The results were expressed as IC_50_ of HOCl-induced inhibition of DHR oxidation.

For the evaluation of O_2_^•^^−^, ^•^NO, and HOCl radical inhibition, epicatechin (EPI) and gallic acid (GA) were used as controls.

#### 2.5.3. Enzyme Inhibition Activity

Tyrosinase inhibition was carried out using a “Colorimetric Tyrosinase Inhibitor Detection Kit” (Sigma-Aldrich, USA) where kojic acid (KA) was used as inhibition control. The control result was expressed as % inhibition.

Inhibition of enzyme elastase was performed following the method of Pinto et al. [30] with slight modifications. The p-nitroaniline released was measured because of the hydrolysis of the substrate MeOSuc-Ala-Ala-Pro-Val-pNa by elastase. The amount of p-nitroaniline was determined at an absorbance of 405 nm. As a control for elastase inhibition, elastatinal (ELA) was used. The control result was expressed as % inhibition.

The HYALase inhibition assay was performed using the method of Nema et al. [31] with some modifications. The assay is based on measuring the decrease in transmitted light intensity due to the presence of particles derived from the digestion of hyaluronic acid (HYAL) by the HYALase. The absorbance was measured at 600 nm. As a control for HYALase inhibition, EPI and GA were used. The results of the EPI and GA controls were expressed as IC_20_.

The inhibition of collagenase enzyme was carried out following the methodology conducted by Kumar et al. [31]. This method is based on the colorimetric measurement of the FALGPA substrate after its degradation by the enzyme. The absorbance was measured at 335 nm. As a control for collagenase inhibition, phenanthroline (PHE) was used. The control result was expressed as IC_50_.

The XO inhibition assay was performed using “Cayman’s Xanthine Oxidase Fluorometric Assay Kit” (Cayman Chem., Ann Arbor, MI, USA). For XO inhibition, EPI was used as a control. The control result was expressed as IC_50_.

For mango peel extract, the results were expressed as IC_50_ at different extract concentrations. All measurements were performed in triplicate.

### 2.6. Study of the Effect on Pigmentation in Zebrafishes

The effect of the mango peel extract on the pigmentation phenotype in zebrafish larvae was evaluated. All experiments were conducted using zebrafish (*Danio rerio*). Adult zebrafish and their offspring were maintained at 27 ± 1 °C for a cycle of 14 h of light and 10 h of darkness. Fertilized eggs develop as “see-through” embryos inside a transparent envelope called the chorion. This acellular layer prevents polyspermy and gives protection to the embryo until approximately 72 h post-fertilization (hpf), when the chorion naturally softens and breaks, releasing the living embryo.

#### 2.6.1. Mango Peel Extract Preparation

The mango peel extract was dissolved in 100% DMSO to prepare a 75,000 ppm stock solution. Larvae were exposed to 150, 75, 50, 50, 10, and 5 ppm mango peel extract.

#### 2.6.2. Preparation and Treatment of Zebrafish Embryos

Fertilized embryos were collected in petri dishes and after 6 hpf, abnormal or not fertilized embryos were discarded. At 22 hpf, mango peel extract, negative control (0.2% DMSO), and positive control (0.003% phenylthiourea (PTU)) were added. Embryos were grown until 96 h post-fertilization.

#### 2.6.3. Pigmentation Assessment

At 6 hpf, abnormal or not fertilized embryos were discarded, and 16 healthy embryos per condition were collected and exposed individually to 5 mango peel extract concentrations plus controls. Embryos were incubated with the drugs for up to 96 hpf. At this time point, after assessing mortality, larvae were imaged using an automated VAST system, and dorsal pigmented area and total body area were assessed. Results were expressed as the pigmented area ratio, which corresponded to the pigmented area divided by the total area of the fish.

#### 2.6.4. Melanin Quantification

At 6 hpf, abnormal or not fertilized embryos were discarded, and 3 replicates of 50 healthy embryos each per condition were pooled and exposed individually to 5 mango peel extract concentrations, plus negative control, and the positive control. Embryos were incubated for up to 96 h post-fertilization, and after assessment of mortality, melanin was quantified spectrophotometrically. The results were expressed as a pigmentation ratio, which corresponded to the amount of melanin in each pool divided by the mean amount of melanin in the negative controls.

### 2.7. Cosmetic Formulation and Stability Tests

#### 2.7.1. Cosmetic Formulation

The formulation of a cosmetic prototype in a gel form was carried out using Unguator EMP equipment (Microcaya, Vizcaya, País Vasco). The following ingredients were used: water, xanthan gum, ethanol, glycerine, and citric acid. Table 1 shows the list of ingredients with their respective INCI name, CAS number, and cosmetic function. Once the formula was obtained, the mango peel extract was incorporated in different percentages (0.5%, 1% and 2%). The gel without extract was used as a negative control. Three batches were prepared, each batch containing the four gels at different percentages of extract. One batch was intended for accelerated stability testing and the two others for long-term stability testing.

To prepare the gel, the xanthan gum was first hydrated. For this purpose, the xanthan gum was slowly sprinkled over water at 60 °C and mixed using a magnetic stirrer. To the mixture of xanthan gum and water, ethanol and glycerine were added, and stirring was continued until a perfect mixture without lumps was achieved. On the other hand, citric acid was dissolved in water. Finally, the citric acid dissolved in water was added to the well-mixed gel without lumps. To obtain the gel, the mixture of ingredients was taken to the Unguator EMP equipment (Microcaya, Vizcaya, País Vasco). In the Unguator, gel formation was carried out in two phases: in the first phase, or wetting phase, the ingredients were mixed at 2150 rpm for 30 s. In the second phase, or swelling phase, the ingredients were mixed at 600 rpm for 9 min and 30 s. The same steps were followed for the incorporation of the mango peel extract into the gel. The extract was previously dissolved in water and ethanol, added to the mixture, and taken to the Unguator. The water content was adjusted according to the extract concentration. The Ph of the gel was adjusted with the addition of citric acid. Ethanol contributed to the stability and shelf life of the cosmetic formulation [32].

#### 2.7.2. Stability Study

Accelerated stability tests evaluated the gel stability under extreme conditions. The methodology described above was used but with minor modifications [33]. The accelerated stability was evaluated by a centrifugation method using a Rotofix 32 A centrifuge (Hettich Zentrifugen, Tuttlingen, Germany) for 30 min at 3000 rpm and room temperature. With the temperature ramp, possible instabilities of the gel were evaluated by subjecting it to a progressive increase in temperature using an oven. An initial temperature of 20 °C was set, increasing every 30 min until a final temperature of 80 °C was reached. The stability of the gels was also evaluated by alternating hot and cold cycles. The gels were kept for 24 h at 60 °C and then placed at 4 °C for a further 24 h. A total of six cycles were performed. All these methods were carried out to observe possible losses in gel stability, such as sedimentation or phase separation.

The long-term stability of the gels was evaluated over a period of three months. For this purpose, the samples were kept for three months in the light and three months in the dark at room temperature. Physicochemical measurements were performed once a month during the three months to observe the evolution of the stability and possible long-term changes in the gels.

Viscosity, color, Ph, and TPC values of the gels were determined after subjecting them to each of the stability methods to check for possible physicochemical modifications. Viscosity measurement was performed at room temperature with an IKA ROTAVISC me-vi Complete viscometer (IKA Designed for Scientist, Staufen, Germany) with SP11 spindle and at two different speeds (10 rpm and 70 rpm). Values were recorded after 60 s of operation. Viscosity was expressed in Pa·s. For color measurement, the values of the colorimetric parameters L*, a*, and b* were determined using a Lovibond TR 500/520 Series colorimeter (Lovibond Tintometer Group, Amesbury, England). For pH measurement, 1 mL of gel was taken and diluted in water at a 1:10 (*v*:*v*) ratio at room temperature using a Fisherbrand™ Accumet™ Portable AP110 pH meter (Thermo Fisher Scientific, Waltham, MA, USA). The TPC of the gels was determined using the Folin–Ciocalteu assay described above with some modifications. To study the stability of cosmetic formulations, all measurements were performed in triplicate.

The physicochemical ranges established to evaluate the stability of the gel formulation were a viscosity between 1 and 100 Pa·s to consider the formulation as a gel and a pH range between 4 and 7 for adequate use on the skin [34,35,36].

### 2.8. Statistical Analysis

To study the stability of cosmetic formulations, all tests were performed in triplicate. Differences in formulations during stability were analyzed using one-way analysis of variance (ANOVA). The statistical significance of the difference between the data pairs was considered significant at *p* < 0.05. This analysis was carried out using the IBM Statistical Package for Social Science (SPSS 15.0) program.

## 3. Results and Discussion

### 3.1. Characterization of Mango Peel Extracts by HPLC-ESI-qTOF-MS

**Figure 1:**
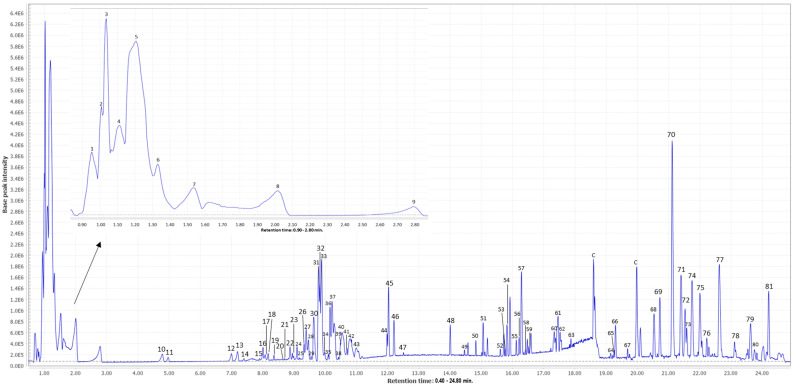
Base peak chromatogram from mango peel extract; C: contaminant (signal detected also in blank samples).

After HPLC-ESI-qTOF-MS analysis, a total of 81 signals were obtained of which 71 were tentatively identified. Figure 1 shows the base peak chromatogram. Table 2 shows the list of all annotated compounds in order of elucidation together with their retention times, *m*/*z* (experimental and theoretical), error (ppm), level of annotation, molecular formula, proposed compounds, and MS/MS fragments.

Among the compounds observed, phytochemicals belonging to the families of organic acids, phenolic compounds, terpenoids, iridoids, aliphatic compounds, carbohydrates, and phospholipids have been detected. In addition, there is a high presence of fatty acids. As can be seen, the main phenolic groups present in mango peel were gallates and gallotannins. These metabolites are produced by various plant species and have been associated with beneficial health effects due to their anti-inflammatory and antioxidant properties [20]. Chromatogram examination of mango peel extract revealed the presence of compounds such as gallic acid, ethyl gallate, ethyl digallate, galloyl-glucose, digalloyl-glucose, trigalloyl-glucose, tetragalloyl-glucose, pentagalloyl-glucose, hexagalloyl-glucose, and hydroxycinnamoyl galloyl glucopyranoside. These compounds have been previously described in mango peel [22,37]. Another important group detected in mango peel extract was quercetin and glycosylated quercetin derivatives such as quercetin and reynoutrin. The analysis confirmed the presence of ellagic acid in mango peel. In addition, the presence of organic acids such as quinic acid and citric acid was also revealed.

**Table 2:** Annotation of phytochemical compounds in mango peel extracts by HPLC-ESI-qTOF-MS.

Peak	RT (min)	*m/z* Experimental [M-H]^−^	*m/z* Theoretical [M-H]^−^	Error (ppm)	Level of Annotation	Molecular Formula	Proposed Compound	MS/MS Fragments	References
**1**	0.96	179.0559	179.0561	−1.18	2	C_6_H_12_O_6_	Glucose	71,89	[38]
**2**	1.01	165.0406	165.0404	1.14	2	C_5_H_10_O_6_	Arabinonic acid	75,165	[39]
**3**	1.04	191.0553	191.0561	−4.25	2	C_7_H_12_O_6_	Quinic acid	78,85,87,111,158	[38]
**4**	1.12	133.0145	133.0142	2.17	2	C_4_H_6_O_5_	Malic acid	71,89,115,133	[38]
**5**	1.20	191.0215	191.0197	9.09	2	C_6_H_8_O_7_	Citric acid isomer 1	57,85,87,111,191	[40]
**6**	1.33	191.0199	191.0197	0.72	2	C_6_H_8_O_7_	Citric acid isomer 2	57,85,87,111,191	[40]
**7**	1.53	331.0680	331.0670	2.98	2	C_13_H_16_O_10_	Galloyl glucose isomer 1	169,125,271	[21]
**8**	2.01	169.0141	169.0142	−0.66	2	C_7_H_6_O_5_	Gallic acid	79,125,169	[21]
**9**	2.80	331.0689	331.0670	5.70	2	C_13_H_16_O_10_	Galloyl glucose isomer 2	79,125,169	[21]
**10**	4.79	483.0787	483.0780	1.42	2	C_20_H_20_O_14_	Digalloyl glucose	125, 169, 331, 483	[21]
**11**	4.97	443.1923	443.1923	−0.02	2	C_21_H_32_O_10_	Dihydrophaseic acid glucoside isomer 1	59,71,89,101,443	[41]
**12**	7.00	517.2289	517.2291	−0.36	2	C_24_H_38_O_12_	Vomifoliol xylosyl glucoside	386,517	[42]
**13**	7.20	519.2463	519.2463	3.01	2	C_24_H_40_O_12_	Platanionoside C	59,89,387	CNP0116765
**14**	7.39	375.1669	375.1660	2.37	2	C_17_H_28_O_9_	Oxo-glucopyranosyloxy-undecenoic acid	329,375	CNP0103002
**15**	7.90	635.0891	635.0890	0.14	2	C_27_H_24_O_18_	Trigalloylglucose isomer 1	125,169,465,483,635	[40]
**16**	8.01	443.1927	443.1922	1.10	2	C_21_H_32_O_10_	Dihydrophaseic acid glucoside isomer 2	59,443	[41]
**17**	8.08	197.0457	197.0455	0.95	2	C_9_H_10_O_5_	Ethyl gallate	78,124,125,169,197	[21]
**18**	8.18	261.1344	261.1343	0.34	2	C_12_H_22_O_6_	Phaseolate	243,261	HMDB0031897
**19**	8.36	403.1610	403.1609	0.22	2	C_18_H_28_O_10_	Glucopyranosyloxy dimethyl decadienedioic acid	59,197,241,403	CNP0365241
**20**	8.65	635.0884	635.0890	−0.96	2	C_27_H_24_O_18_	Trigalloylglucose isomer 2	125,169,331,465,483,635	[40]
**21**	8.70	417.1758	417.1766	−1.95	2	C_19_H_30_O_10_	Hydroxipropyl methoxyphenox phenoxy propanediol glucoside	417	HMDB0040353
**22**	8.88	477.1037	477.1038	−0.23	2	C_22_H_22_O_12_	Hydroxycinnamoyl galloyl glucopyranoside isomer 1	119,163,313, 477	HMDB0039190
**23**	8.96	477.1036	477.1038	−0.44	2	C_22_H_22_O_12_	Hydroxycinnamoyl galloyl glucopyranoside isomer 2	119,163,477, 313	HMDB0039190
**24**	9.11	787.0989	787.0999	−1.29	2	C_34_H_28_O_22_	Tetragalloylglucose isomer 1	125,169,465, 617,635	[21]
**25**	9.15	425.1779	425.1817	−9.04	2	C_21_H_30_O_9_	Abscisic acid glucopyranosyl ester	147,153,263, 425	PubChem: 102173239
**26**	9.32	503.2463	503.2498	−7.00	2	C_24_H_40_O_11_	Megastigmadienediol apiosyl glucoside	101,161,371, 503	HMDB0029766
**27**	9.39	787.0972	787.0999	−3.45	2	C_34_H_28_O_22_	Tetragalloylglucose isomer 2	125,169,465, 617,635	[21]
**28**	9.46	787.0897	787.0999	−12.97	2	C_34_H_28_O_22_	Tetragalloylglucose isomer 3	125,169,465, 617, 635	[21]
**29**	9.55	787.0997	787.0999	−0.27	2	C_34_H_28_O_22_	Tetragalloylglucose isomer 4	125,169,465, 617, 635	[21]
**30**	9.65	300.9988	300.9990	−0.70	2	C_14_H_6_O_8_	Ellagic acid	242,257, 270,273,300	[21]
**31**	9.79	463.0886	463.0882	0.84	2	C_21_H_20_O_12_	Quercetrin isomer 1	151,178,255, 271,300	[21]
**32**	9.84	463.0890	463.0882	1.65	2	C_21_H_20_O_12_	Quercetrin isomer 2	151,178,255, 271,300	[21]
**33**	9.89	939.1012	939.1109	−10.34	2	C_41_H_32_O_26_	Pentagalloyl glucose isomer 1	125,151,169,617,939,769,787	[21]
**34**	9.66	939.1057	939.1109	−5.55	2	C_41_H_32_O_26_	Pentagalloyl glucose isomer 2	125,151,169,617,769,787,939	[21]
**35**	10.11	655.2623	-	-	4	-	Unknown	-	-
**36**	10.16	433.0761	433.0776	−3.49	2	C_20_H_18_O_11_	Reynuotrin	271,300,301,433	[40]
**37**	10.23	1091.1130	1091.1218	−8.08	2	C_48_H_36_O_30_	Hexagalloyl glucose isomer 1	169,431,617,769,939	[40]
**38**	10.43	447.0915	447.0933	−4.05	2	C_21_H_20_O_11_	Quercetrin isomer 3	271,151,301,447	[21]
**39**	10.49	491.2120	491.2134	−2.87	2	C_22_H_36_O_12_	Jasminoside I	313,445,491	CNP0216715
**40**	10.55	621.0587	-	-	4	-	Unknown	-	-
**41**	10.70	349.0558	349.0565	−2.04	2	C_16_H_14_O_9_	Ethyl digallate	124,125,169, 197	[21]
**42**	10.81	697.0682	-	-	4	-	Unknown	-	-
**43**	11.00	773.0707	-	-	4	-	Unknown	-	-
**44**	11.99	327.2171	327.2177	−1.87	2	C_18_H_32_O_5_	Corchorifatty acid F isomer 1	171,211,327	HMDB0035919
**45**	12.03	327.2180	327.2177	0.88	2	C_18_H_32_O_5_	Corchorifatty acid F isomer 2	171,211,327	HMDB0035919
**46**	12.21	301.0349	301.0354	−1.70	2	C_15_H_10_O_7_	Quercetin	151,178	[43]
**47**	12.51	329.2332	329.2333	−0.34	2	C_18_H_34_O_5_	Trihydroxy octadecenoic acid	171,211,229,311,329	HMDB0030936
**48**	14.01	293.2114	293.2122	−2.77	2	C_18_H_30_O_3_	Hydroxyoctadeca trienoic acid isomer 1	59,195,275,235, 293	HMDB0010203
**49**	14.46	309.2065	309.2071	−2.17	2	C_18_H_30_O_4_	Hydroperoxyoctadeca trienoic acid	171,185,209, 291,293,309	CNP0346715
**50**	14.83	675.3614	675.3597	2.48	3	C_33_H_56_O_14_	Gingerglycolipid A isomer 1	397,415,675	[44]
**51**	15.07	675.3618	675.3597	3.01	3	C_33_H_56_O_14_	Gingerglycolipid A isomer 2	397,675	[44]
**52**	15.61	564.3312	-	-	4	-	Unknown	-	-
**53**	15.77	476.2787	476.2783	0.84	3	C_23_H_44_NO_7_P	Octadecadienoyl lysophosphatidyl ethanolamine isomer 1	279,476	HMDB0011507
**54**	15.82	293.2114	293.2122	−2.77	2	C_18_H_30_O_3_	Hydroxyoctadeca trienoic acid isomer 2	171,235,275, 293	HMDB0010203
**55**	16.12	699.3818	-	-	4	-	Unknown	-	-
**56**	16.22	452.2786	452.2782	0.86	2	C_21_H_44_NO_7_P	Hexadecanoyl lysophosphatidyl ethanolamine isomer 2	255,452	HMDB0011473
**57**	16.29	540.3306	-	-	4	-	Unknown	-	-
**58**	16.42	725.3986	725.4022	−4.98	2	C_34_H_62_O_16_	Dioleic glucoside	281,397,415,679,725	[40]
**59**	16.53	295.2279	295.2278	0.30	2	C_18_H_32_O_3_	Coriolic acid	183,119,277, 295	HMDB0062652
**60**	17.34	633.3799	633.3797	0.61	2	C_39_H_54_O_7_	hydroxypyracrenic acid isomer 1	145,633	HMDB0029780
**61**	17.46	633.3801	633.3797	0.30	2	C_39_H_54_O_7_	hydroxypyracrenic acid isomer 2	145,633	HMDB0029780
**62**	17.53	471.3488	471.3480	1.67	2	C_30_H_48_O_4_	Maslinic acid	99,393,471	[45]
**63**	17.87	445.3170	445.3171	−0.26	2	C_24_H_46_O_7_	Monogalactosyl stearate	59,281,445	CNP0179792
**64**	19.16	617.3867	617.3847	3.22	2	C_39_H_54_O_6_	Coumaroylalphitolic acid	117,145,617	HMDB0036299
**65**	19.26	299.2596	299.2591	1.63	2	C_18_H_36_O_3_	Hydroxyoctadecanoic acid	59,299	HMDB0112182
**66**	19.30	279.2334	279.2329	1.75	2	C_18_H_32_O_2_	Linoleic acid	97,219,279	[40]
**67**	19.75	343.2660	344.2720	4.98	2	C_23_H_36_O_2_	Benzoyl hexadecanone	81,343	HMDB0035582
**68**	20.54	345.2801	345.2799	0.54	2	C_23_H_38_O_2_	Heptadecenyl benzenediol	81,125,303,345	HMDB0038527
**69**	20.72	981.5788	981.5904	−11.91	2	C_51_H_86_N_2_O_16_	Isovalerylspiramycin Iii	277,935,981	CNP0328040
**70**	21.12	621.4392	621.4372	3.20	2	C_36_H_62_O_8_	Ginsenoside RH2	59,161,621	[46]
**71**	21.40	758.5544	759.5572	6.54	2	C_45_H_78_NO_6_P	(Geranylgeranyl)-Sn-Glycero-Phophoethanolamine isomer 1	59,89	CNP0350471
**72**	21.53	758.5428	759.5572	−8.75	2	C_45_H_78_NO_6_P	(Geranylgeranyl)-Sn-Glycero-Phophoethanolamine isomer 2	59,89	CNP0350471
**73**	21.59	429.3033	429.3010	5.19	2	C_27_H_42_O_4_	Plastoquinone 1	133,135,429	CNP0152651
**74**	21.75	819.5288	819.5264	2.88	2	C_45_H_74_O_10_	Diethyl tetramethyl propanyl octaoxapentacyclotetracontane tetrone	277,513,773	CNP0271090
**75**	22.00	959.5959	-	-	4	-	Unknown	-	-
**76**	22.22	795.5314	-	-	4	-	Unknown	-	-
**77**	22.29	431.3184	431.3167	3.94	2	C_27_H_44_O_4_	Caffeic acid stearyl ester isomer 1	133,135,161,179,431	CNP0292000
**78**	22.64	431.3182	431.3167	3.45	2	C_27_H_44_O_4_	Caffeic acid stearyl ester isomer 2	133,1351,61,179,431	CNP0292000
**79**	23.12	797.5463	-	-	4	-	Unknown	-	-
**80**	23.63	489.3605	489.3585	4.06	2	C_30_H_50_O_5_	Escinidin	57,125,489	HMDB0034525
**81**	24.21	545.2950	546.2986	7.52	2	C_34_H_42_O_6_	Dimethyloctadienyl trihydroxy-methoxy xanthenone	369,527,545	PubChem: 49798966

RT: retention time.

### 3.2. Evaluation of Total Phenolic Content and Antioxidant Capacity

The TPC of mango peel extract was determined using the Folin–Ciocalteu method. The values obtained in the assay are shown in Table 3. Several reports indicate that mango fruit contains considerable amounts of phenolic compounds, the content being higher in the peel than in the pulp [47,48].

This TPC value was compared with TPC values from other studies. In this sense, mango peel extract showed a higher TPC value than reported by Rojas-Bravo et al. (from 20 to 32 mg GAE/g DE) and Martínez-Ramos et al. (2.05 mg GAE/g DE) [49,50]. The values obtained are close to the results reported by Alañon et al. in different mango varieties [51]. On the other hand, Umamahesh et al. reported a higher TPC value in different mango cultivars; the highest TPC value was 87.38 ± 0.43 mg GAE per g [52]. The variation in phenolic content can be ascribed to factors like different cultivars, ripening stages, environmental conditions, origin, or various pre-treatment and extraction methods [53].

Regarding antioxidant capacity, bioactive compounds present in plants, such as phenolics, protect cells from endogenous and exogenous oxidative stress by preventing the formation of free radicals and avoiding cell damage [54]. These compounds have shown a high antioxidant power due to their chemical structure and the presence of different functional groups [55]. Therefore, the capacity of the extract might be related to the ability of the compounds to protect the biological system against the harmful effects of the oxidation process [56]. To evaluate in a more detailed way the antioxidant capacity of mango peel, several methodologies have been carried out based on different mechanisms through which phenolic compounds can exert their antioxidant action depending on their structure. HAT reactions are based on the transfer of a hydrogen atom while SET reactions are based on the transfer of an electron [57]. In this work, we have used the ORAC method, which is the capacity of the extract to capture radicals through HAT reactions, and the FRAP and TEAC methods, which evaluate the capacity to neutralize radicals by means of a SET mechanism.

The antioxidant capacity of mango peel could be directly related to the presence of phenolic compounds. Several studies have reported that the content of phenols in plants is associated with their antioxidant actions allowing them to act as reducing agents and hydrogen donors [58].

The FRAP value 0.32 ± 0.79 mmol FeSO_4_/g for mango peel reported by Safdar et al. was lower than that reported in this study [59]. Yu-Ge Liu et al. reported FRAP and TEAC values for mango peel of eight different cultivars (from 0.016 to 0.122 mmol FeSO_4_/g DE and from 58 to 183 µmol TE/g DE, respectively), which were quite similar to ours [60]. Castañeda-Valbuena et al. also reported TEAC values for mango peel between 1 and 4.4 mmol TE/g DE [61]. Sogi et al. reported ORAC values for mango peel that ranged from 0.42 to 0.78 mmol TE/g DE [62]. The different antioxidant values can be affected by multiple factors involved in the recovery of bioactive compounds such as sample preparation, extraction techniques, or the nature of the solvents [63].

### 3.3. Evaluation of ROS/RNS Scavenging Activity

ROS are involved in various physiological functions in the body and are generated as by-products of cellular metabolism. However, environmental factors such as ultraviolet radiation and pollutants greatly increase ROS production, producing an imbalance that leads to cellular and tissue damage [64]. In the skin, an excess of ROS promotes skin aging and inflammation and leads to the appearance of diverse dermatological conditions such as urticaria, atopic dermatitis, psoriasis vulgaris, and acne, among others [65]. In addition, UV hyperpigmentation phenomena are attributed to excessive melanin synthesis promoted by O_2_^•^^−^, H_2_O_2,_ and ^•^NO [64].

To better understand the antioxidant activity of mango peel, an antiradical activity evaluation was carried out. Table 3 shows the results obtained from mango peel after evaluating its free-radical scavenging capacity. Table 3 also shows the results of the positive controls used to compare our results. The IC_50_ value of mango peel for ^•^NO showed no significant difference (*p* > 0.05) to the value of GA (7.5 and 1.4, respectively). The IC_50_ values of mango peel for O_2_^•^^−^ radical and HOCl showed significant differences compared to the IC_50_ values of GA and EPI.

The values obtained showed that mango peel extract possesses a free-radical scavenging capacity, especially against the ^•^NO radical. Considering the compounds previously identified by HPLC-ESI-qTOF-MS in mango peel, it could be said that the free-radical scavenging capacity is determined by its composition. The results may be due to the presence of gallic acid and gallotannins, which have shown gallic acid to be one of the main natural compounds with high antiradical activity [66]. It should also be noted the presence of quercetin and quercitrin, both of which have remarkable bioactivity against species such as O_2_^•^^−^ and ^•^NO [67]. Yin et al. also reported the protective power of quercitrin against oxidative damage induced by UVB exposure [68].

### 3.4. Enzyme Inhibition Activity

Skin aging involves a progressive loss of skin elasticity and resilience, leading to the appearance of wrinkles and other visible signs of aging.

The extracellular matrix (MEC), located in the dermis, is composed of fibroblasts and protein fibers composed of collagen and elastin. Collagen provides the skin’s strength and flexibility while elastin provides elasticity to the skin and hyaluronic acid (HA) helps to retain moisture in the skin. As a consequence of aging, a decrease in collagen, elastin, and HA levels occurs through different mechanisms [69]. These mechanisms include the enzymatic degradation of collagen and elastin fibers and the attack of fibroblasts by free radicals, which are responsible for the synthesis of collagen, elastin, and HA. Bioactive compounds that are capable of inhibiting collagenase, elastase, and HYALase enzymes, responsible for the degradation of collagen, elastin, and HA, will have an anti-aging effect on the skin, improving its elasticity and structure and maintaining the integrity of the MEC [70].

One sign of photoaging is hyperpigmentation. Melanin is responsible for pigmentation in the skin and has a beneficial effect on the photoprotection of the skin against UV radiation damage [71]. However, excessive production of melanin causes hyperpigmentation in the skin leading to spots and melasma. The enzyme responsible for melanin synthesis is tyrosinase. Since tyrosinase is the enzyme in charge of the melanogenesis process, its inhibition makes it a target for study as a possible lightening effect [71].

To learn more about mango peel extract as an antioxidant and anti-aging strategy, its ability to inhibit tyrosinase, elastase, HYALase, collagenase, and XO enzymes was determined. Table 3 shows the IC_50_ values of mango peel extract for each enzyme.

Mango peel extract showed high anti-HYALase activity compared to EPI and GA controls. Kim et al. isolated the compound 1,2,3,4,6-penta-O-galloyl-β-D-glucose (PGG) which showed significant elastase and HYALase inhibitory activities with IC_50_ values of 57 μg/mL and 0.86 mg/mL, respectively. Furthermore, PGG treatment in rabbit articular chondrocytes induced the expression of type II collagen. In HPLC-ESI-qTOF-MS analysis of mango peel extract, the presence of PGG was qualitatively determined, which could significantly influence the values obtained [72]. In the case of tyrosinase, elastase and collagenase enzymes, the inhibitory capacity of mango peel extract was lower than that of the control.

Previous studies established that compounds such as flavonoids and phenolic acids are capable of inhibiting enzymes through the interaction of their hydroxyl group with the functional group of the enzyme. Flavonoids will induce changes in the secondary structure of enzymes and consequently lead to changes in their activity [73]. Mango peel contains phenolic compounds including gallic acid and hydrolysable tannins, which are known to be metal chelators and therefore, can bind to the active site and prevent the substrate from being enzymatically hydrolyzed [74]. Therefore, the inhibitory activity of mango peel extract could again be explained by the presence of phenolic compounds. For example, quercetin was found to exhibit anti-tyrosinase activity by chelating copper from its active site due to the catechol group of quercetin [75]. Zeng et al. studied the binding of different flavonoids to the enzyme HYALase and observed that quercetin was able to bind spontaneously with the enzyme by means of electrostatic forces, hydrophobic interaction, and hydrogen bonding [73,76].

Regarding the inhibition of the XO enzyme, similar IC_50_ values were obtained for mango peel extract and epicatechin, thus confirming their inhibition potential against the XO enzyme. Previous reports have highlighted phenolic compounds as potential therapeutic agents due to their essential role in XO inhibition. Gallic acid and gallotannins present in mango have been reported to have great potential against XO activity [77,78]. Barreto et al. previously reported penta-O-galloyl-glucoside as one of the main compounds detected in mango peel and seed with XO inhibitory capacity [22]. Therefore, the phenolic content in mango peel extract is directly related to its ability to inhibit the XO enzyme.

Ochocka et al. studied mangiferin and its inhibition capacity on elastase and collagenase with IC_50_ values of 59 mg/L and 107 mg/L, respectively [79]. Shi et al. reported the inhibitory capacity of mango leaves against tyrosinase with an IC_50_ value of 18 mg/L [80]. Namngam et al. examined the anti-tyrosinase and anti-HYALase activity of mango seed with IC_50_ values of 20 mg/L and 37 mg/L, respectively [81].

After a thorough review of the literature, it is evident that there is a remarkable paucity of information regarding the enzyme-inhibitory capacity of mango peel extract. The anti-enzymatic capacity of mango peel was compared with those of other tropical fruits: avocado peel reported an anti-collagenase IC_50_ value of 81 mg/L [82]. Custard apple peel extract reported an IC_50_ value of 4.4 for XO enzyme inhibition ability, higher than the value obtained for mango peel [23]. Therefore, the values obtained are similar to the results found in the literature. Mango peel extract showed potent anti-tyrosinase, anti-HYALase, anti-collagenase, and anti-XO activities.

### 3.5. Study of the Effect on Pigmentation in Zebrafish

Melanin is synthesized from a process called melanogenesis, which is a rather complex metabolic pathway that combines spontaneous chemical and enzymatically catalyzed reactions [83]. Several studies have focused on the prevention of depigmentation or hyperpigmentation in the skin by regulating the process of melanogenesis [84]. In this context, zebrafish have been used extensively to assess in vivo the pigmenting or depigmenting activity of melanogenic regulatory compounds. Melanin pigments can be observed on the surface of the zebrafish allowing observation of the pigmentation process.

**Figure 2:**
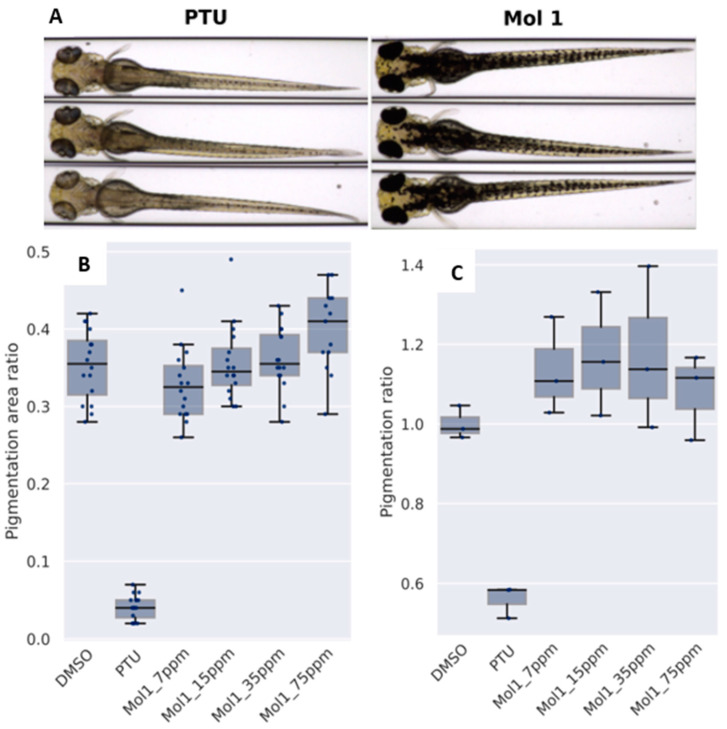
(**A**) Representative images of larval pigmentation at 96 h post-fertilization. (**B**) Pigmentation analysis at 96 h post-fertilization. Each dot represents individual larvae. (**C**) Melanin quantification analysis at 96 h post-fertilization. Each point represents a set of 50 larvae. DMSO: negative control; PTU: 1-phenyl 2-thiourea as a positive control; Mol1: mango peel extract.

Zebrafish embryos were treated with mango peel extract at different concentrations. Analysis revealed that the mango peel extract had a detectable pigmenting effect on the pigmentation pattern and therefore did not induce depigmentation. However, measurement of the total melanin content showed that the mango peel extract had no effect on the amount of melanin produced after 96 h of treatment. In addition, the cytotoxic effect of the extract was also evaluated. Embryos treated with mango peel extract showed 100% mortality at 150 ppm. Figure 2 shows representative images of larval pigmentation, pigmentation analysis, and melanin quantification analysis at 96 h post-fertilization.

Therefore, mango peel extract did not show depigmenting properties, although, it is possible that it induced an increase in pigment, as significant differences in pigmentation were detected at 75 ppm. To date, there is little literature on the effect of mango extract on zebrafish pigmentation. However, the ability of some phenolic compounds on zebrafish pigmentation has been previously reported. For example, gallic acid was able to strongly reduce pigmentation in zebrafish embryos by up to 40% at a concentration of 40 µM [85]. In addition, several studies have also observed the ability of the ginsenoside Rb2 to inhibit melanin synthesis in zebrafish in a dose-dependent manner [86]. Both of these compounds were detected in mango peel extract.

### 3.6. Cosmetic Formulation and Stability Tests

In view of the potential of the mango peel extract and its phytochemical composition, the extract was incorporated into a cosmetic prototype at different percentages for its vehiculation.

#### 3.6.1. Evaluation of Physicochemical Parameters and TPC of Zero-Time Formulations

Initially, the physicochemical parameters such as viscosity, color, and pH of the gel containing no extract (0%) and mango peel extract (0.5%, 1%, and 2%) were determined. In addition, the TPC was evaluated by the Folin–Ciocalteu method in all formulations. Table 4 shows all the measured parameters at zero time.

The presence of xanthan gum and glycerine was decisive in obtaining the gel and had a significant influence on its viscosity. For a product to be considered a gel, its viscosity must be in the range of 1 and 100 Pa·s, and the thickening agents in the gel must be below 10% [34]. In the present work, the viscosity of the gels was measured at different speeds (10 rpm and 70 rpm). The viscosity values at 10 rpm ranged from 8.40 to 13.70 Pa·s while the viscosity values at 70 rpm ranged from 1.60 to 2.50 Pa·s. Therefore, the gel formulations in this work exhibited adequate viscosity ranging between 1 and 100 Pa·s. In addition, it was observed that increasing the speed caused a decrease in viscosity values, showing a non-Newtonian rheological behavior in all gels, which is a pseudoplastic compartment. The pseudoplastic behavior in cosmetic formulations allows a better dispersion on the skin [87]. The inclusion of the extract caused an increase in viscosity in the base gel. In fact, the increase in viscosity was proportional to the extract concentration. Ahshawat et al. carried out the preparation of herbal creams at different extract concentrations and observed that as the extract concentration increased, the viscosity of the cream increased [88]. Nunes et al. carried out the incorporation of by-products of the olive oil industry in creams and similarly observed that viscosity increased with the addition of the extract [89].

To evaluate the color of the gels, the CIELab space was used. The CIELab space is a uniform three-dimensional space determined by three color coordinates L*, a*, and b* [90]. In addition, CIELab space has been used by different authors for the study of color in food and cosmetics [91,92]. The parameter L* is the vertical axis and indicates the brightness; it has a range from 0 to 100, where L* = 0 completely opaque and L* = 100 transparent. Considering this scale, the L* parameter slightly decreased as the percentage of extract increased. The parameters a* and b* are the perpendicular horizontal axes and define red to green and blue to yellow, respectively. The parameter a* lies between the colors red and green and ranges from −100 to +100. Positive a* values indicate red color while negative a* values indicate green color [90]. All gels showed negative a* values indicating the prevalence of a slight green color. There was little variation in a* values after the addition of the extract. However, the gel with the highest green color was the one containing 0.5% extract with a difference of −0.53 units. The parameter b* lies between the colors yellow and blue and ranges from −100 to +100. Positive values of b* indicate yellow color while negative values of b* indicate blue color [90]. All gels showed positive b* values, indicating the presence of yellow color. The addition of the extract to the base gel caused an increase of approximately five units in the b* values. Gels with 0.5%, 1%, and 2% extract showed similar b* values. For example, Censi et al. developed a cream with açai extract as a bioactive ingredient and also observed a progressive decrease in the L* parameter with increasing açai extract content [93].

In the field of cosmetics, the natural pH of the skin plays a fundamental role in the development of formulations. The skin shows an acidic surface known as ‘’acid mantle’’ and follows a strong gradient through the stratum corneum, which is important for the control of different enzymatic activities and skin renewal [94]. In this regard, all formulations were within the range considered suitable for topical products, between 4 and 7, with the ideal pH for the face and body being approximately 4.7 [35,36]. The pH decreased as the concentration of mango peel extract increased. The pH reduction could be related to the acidic nature of the bioactive ingredient due to the presence of phenolic compounds, mainly phenolic and organic acids. Pinto et al. determined the pH of the base cream and the cream containing *C. sativa* and observed that the pH of the cream containing the extract was lower than that of the base cream [95].

The TPC of the different gels was measured by the Folin–Ciocalteu method. The results obtained showed an increase in phenolic content as the concentration of mango peel extract in the gels increased, as expected. Salem et al. added grape seed extract of different varieties in cosmetic creams and evaluated their total phenolic content. The cream containing 5% extract had a TPC value of 0.046 mg GAE/mL [96]. Mapaung et al. determined the TPC in 23 functional cosmetic creams and obtained TPC values between 0.015 and 1.59 mg GAE/g of the samples [97]. Censi et al. carried out the development of a cosmetic formulation containing 2% açai extract and a TPC value of 0.029 mg GAE/g sample. The TPC of this lotion was significantly lower than the TPC value of the 2% mango peel extract gel of the present study (1.56 mg GAE/g sample) [93].

#### 3.6.2. Stability Tests

The incorporation of extracts rich in bioactive compounds from natural sources represents a challenge due to the instability of an aqueous system. The physical stability of semi-solid formulations can be affected by factors such as temperature, oxygen, product aging during storage time, and degradation of phenolic compounds [98,99]. By European Regulation No. 1223/2009, the safety and stability of formulations are set as a top priority for the marketing of cosmetic products [100]. Therefore, it was important to guarantee the effectiveness and stability of a topical gel based on mango peel extract through storage under different conditions, evidencing the stability of its ingredients and active ingredients. The evaluation of physicochemical parameters is essential to obtain interesting information about the stability and bioactivity of the final product. In the present work, two types of stability studies were carried out on the gels, a preliminary stability or accelerated stability study and a long-term stability study.

##### Accelerated Stability

Accelerated stability studies provide insight into the potential physical, organoleptic, chemical, or microbiological changes that may occur in cosmetic products when exposed to factors such as temperature, light, humidity, and others [89]. These studies allow adjustments to be made to the formula, determine shelf life, and ensure the safety, performance, and appearance of the product. Viscosity, color, pH, and TPC values obtained from centrifugation, temperature ramp, heating, and cooling cycles were compared with the initial values, and it was observed whether the formulation was a stable vehicle for the extract.

**Figure 3:**
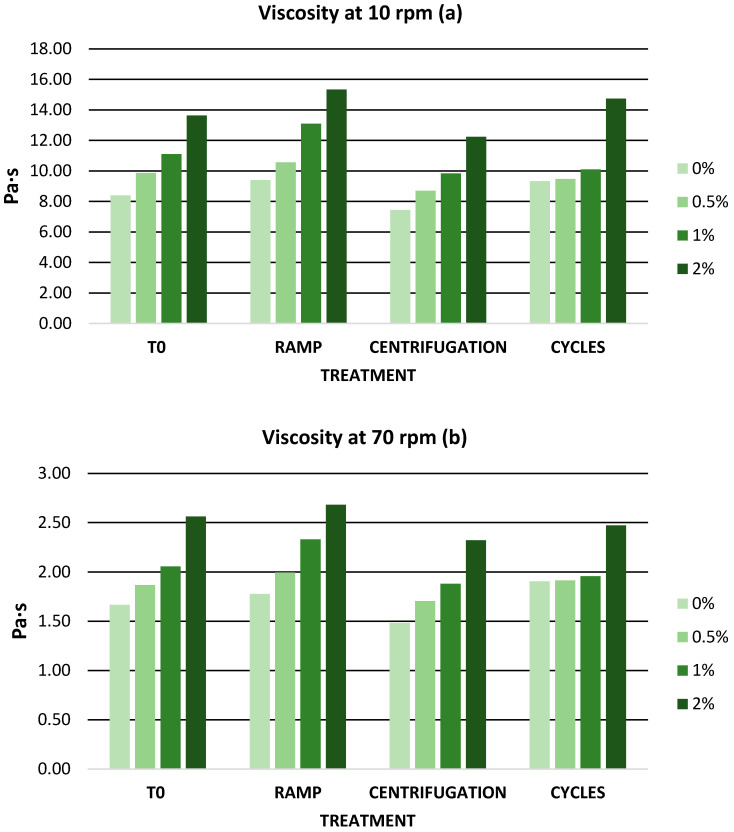
(**a**) Viscosity values at 10 rpm during accelerated stability; (**b**) viscosity values at 70 rpm during accelerated stability. rpm: revolutions per minute; 0%: gel without extract; 0.5%: gel with 0.5% mango peel extract: 1%: gel with 1% mango peel extract; 2%: gel with 2% mango peel extract.

After accelerated stability tests, no phase separation was observed, and all gels maintained a homogeneous appearance. It has been established that emulsion gels exhibit enhanced stability in comparison to emulsions. This is attributed to the ability of emulsion gels to counteract common compatibility issues like creaming, flocculation, or coalescence by augmenting viscosity and establishing a structured aqueous phase network facilitated by gels [101]. In addition, the pseudoplastic behavior of the gel due to the presence of xanthan gum gives it high stability over a wide range of temperatures and ionic strength, which makes it more shear stable [102]. Viscosity evolved similarly during temperature ramping and centrifugation. The process that affected the viscosity values the most was temperature cycling due to the abrupt temperature changes. However, the viscosity values of all gels (Figure 3) remained within the viscosity range for a gel formulation. Chuarienthong et al. prepared cosmetic formulations with rooibos and soybean extract and studied the viscosity changes after temperature cycling. The viscosity changes were 4.4 Pa·s and 4.1 Pa·s for the rooibos and soybean formulations, respectively. The viscosity changes in these formulations were much higher than those obtained in the present study after temperature cycling, where the maximum variation was 1.10 Pa·s [103].

**Figure 4:**
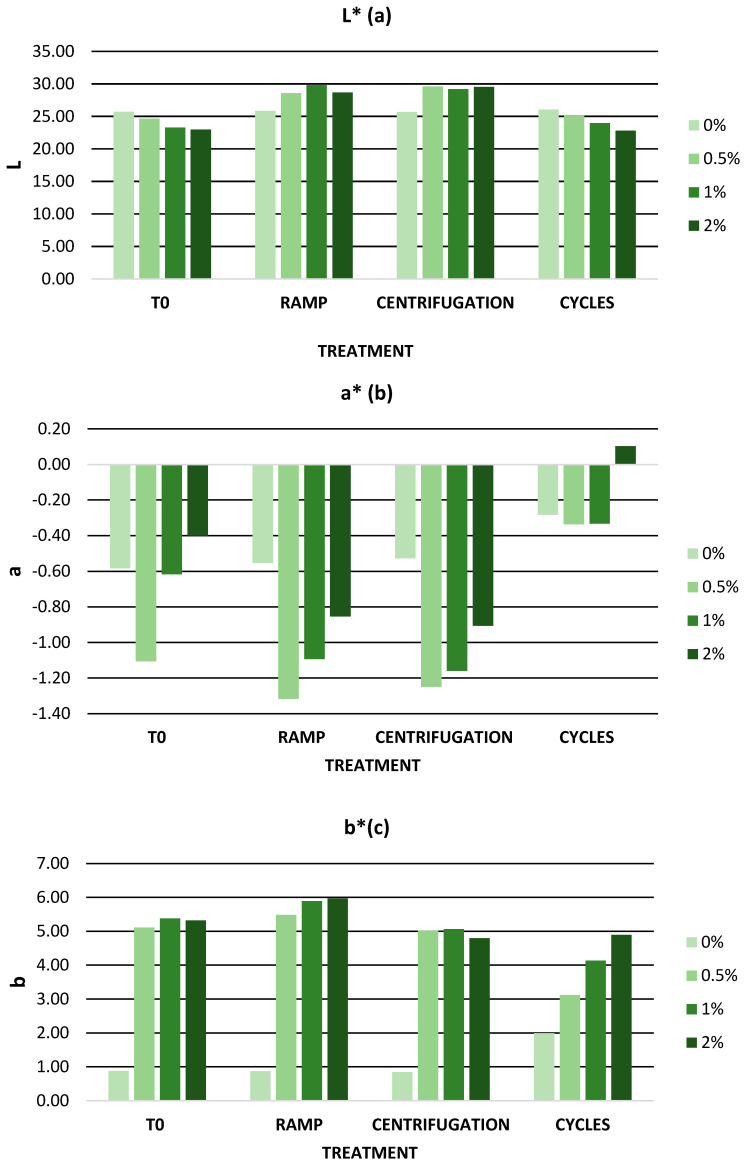
(**a**) parameter L*; (**b**) parameter a*; (**c**) parameter b* during accelerated stability. 0%: gel without extract; 0.5%: gel with 0.5% mango peel extract: 1%: gel with 1% mango peel extract; 2%: gel with 2% mango peel extract.

Figure 4 represents the CIELab values of all gels after accelerated stability. The L*, a*, and b* values showed a variation of less than 10 units with respect to the zero-time values. In Figure 4, we can observe how temperature cycling was the process that most affected the color of the gels. Color variations could be associated with the degradation of phenolic compounds due to sudden temperature changes although it is difficult to correlate CEILAB parameters with this degradation [101]. However, taking into account the ranges of values established in the CIELab space previously described, the variations of L*, a*, and b* were minimal.

**Figure 5:**
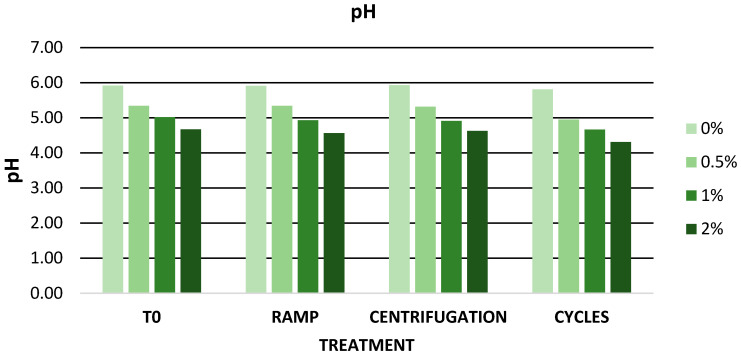
pH values during accelerated stability. 0%: gel without extract; 0.5%: gel with 0.5% mango peel extract: 1%: gel with 1% mango peel extract; 2%: gel with 2% mango peel extract.

Figure 5 shows the pH values during the accelerated stability study. No pH changes affecting the quality of the gels were observed. The pH of all gels is between 4 and 7, indicating biocompatibility with the skin [88,89].

**Figure 6:**
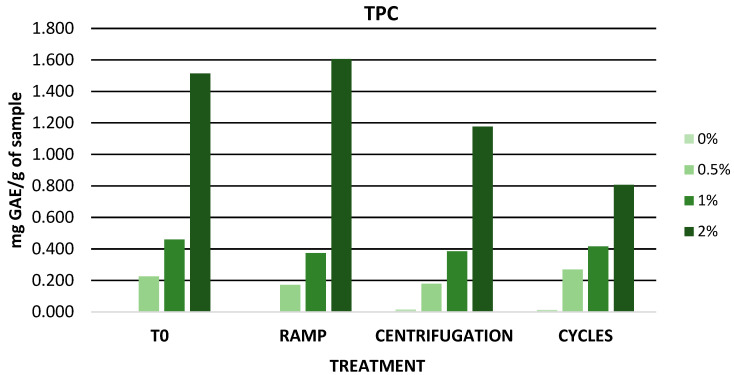
TPC values during accelerated stability. GAE: gallic acid equivalent; 0%: gel without extract; 0.5%: gel with 0.5% mango peel extract: 1%: gel with 1% mango peel extract; 2%: gel with 2% mango peel extract.

Figure 6 shows the TPC values obtained after accelerated stability. The gels with 0.5% and 1% extract showed a constant TPC value throughout the accelerated stability study. However, the gel with 2% extract showed a higher variation in TPC values. These variations could be related to the degradation of the bioactive compounds present in the extract due to the high temperatures to which the formulation was subjected. Maisuthisakul et al. determined the TPC value of a lotion containing mango seed extract and observed that after temperature cycling the TPC value decreased similar to the results of the present study [102].

After accelerated stability, the physicochemical values of the gels remained within the established limits and ranges. It should be noted that the gels with 0.5% and 1% mango peel extract showed higher stability than the gel with 2% extract due to a lower variability of the physicochemical values within the established limits. The most considerable variations occurred during the temperature cycling test. Considering that small variations in a cosmetic product subjected to extreme temperatures are acceptable, it can be considered that the formulations developed in the present work were stable during the accelerated stability evaluation [98]. Furthermore, the formulations showed optimal physicochemical values for use on the skin.

##### Long-Term Stability

The main objective of long-term stability is to determine the shelf life of the cosmetic product [99]. In the present work, the long-term stability study was carried out in a three-month evaluation time with measurements once a month (T0, T1, T2, and T3). During the three months, the samples with 0%, 0.5%, 1%, and 2% mango peel extract were stored in light and dark. The viscosity, color, pH, and TPC values obtained at the different times were compared with the initial values at time zero.

**Figure 7:**
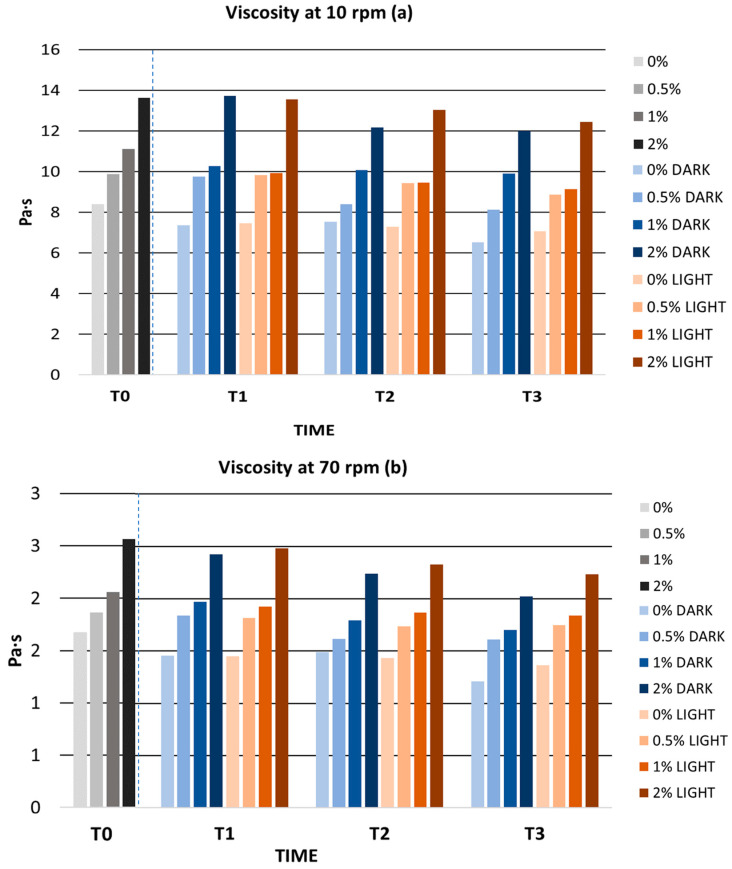
(**a**) Viscosity values at 10 rpm during long-term stability; (**b**) viscosity values at 70 rpm during long-term stability. rpm: revolutions per minute; 0%: gel without extract; 0.5%: gel with 0.5% mango peel extract: 1%: gel with 1% mango peel extract; 2%: gel with 2% mango peel extract; DARK: samples stored in the dark; LIGHT: samples stored in the light. The dashed line indicates the separation between the control and the long-term stability samples.

The long-term stability of the gels was characterized by the absence of phase separation. During storage time, viscosity values decreased in all gels; however, these changes did not affect stability. In Figure 7, we can observe how the decrease in viscosity is directly proportional to time. This decrease in viscosity with time could be related to storage conditions, temperature, and/or thermal properties of xanthan gum and water. Despite this, the viscosity values always remained between 1 and 100 Pa·s, a range in which the cosmetic is considered a gel. In this regard, Plyduang et al. developed a cream with *E. guineensis* extract and measured its viscosity after six months of storage. The cream showed a viscosity variation of 8.14 Pa·s (from 55.41 to 63.55 Pa·s), much higher than that shown by the gels in the present study, whose maximum variation in viscosity was 1.9 Pa·s [100]. Moraes et al. formulated a cream containing 0.4% rutin succinate and observed that after 90 days of storage in indirect light, viscosity variations of up to 20% occurred. The formulation of Moraes et al. showed a much higher viscosity variation compared to the viscosity variations obtained in our work [104].

**Figure 8:**
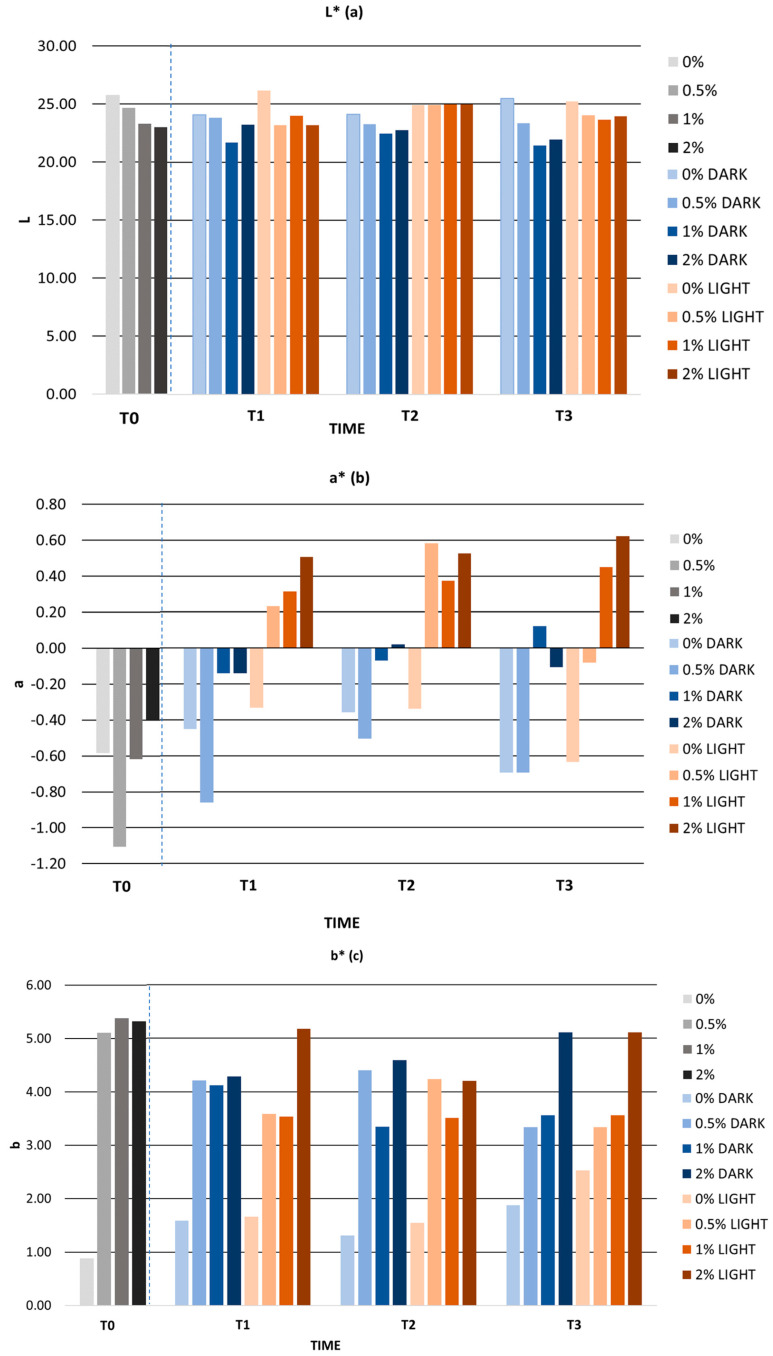
(**a**) parameter L*; (**b**) parameter a*; (**c**) parameter b* during accelerated stability. 0%: gel without extract; 0.5%: gel with 0.5% mango peel extract: 1%: gel with 1% mango peel extract; 2%: gel with 2% mango peel extract; DARK: samples stored in the dark; LIGHT: samples stored in the light. The dashed line indicates the separation between the control and the samples during long term stability.

Both light and dark-stored formulations showed a homogeneous appearance. Figure 8 shows the L*, a*, and b* values during storage of the gels in the light and in the dark. The L* parameter remained constant in all cases and presented a mean variation of 0.82 units in the light and 1.07 units in the dark. The a* parameter varied very slightly. Samples with extract stored under light conditions showed positive a* values from the second month onwards, indicating the presence of red color shades. Despite this, the mean variation in the a* parameter was 0.35 and 0.86 units in the light and in the dark, respectively. The b* parameter decreased in all cases, which was associated with a loss of yellow color. Formulations stored in the light showed a greater variation in parameter b* than samples stored in the dark. However, these variations were minimal, with an average variation of 1.19 units in the light and 1.05 units in the dark. Considering the CIELab scale, the L*, a*, and b* parameters remained stable over time, both in the light and in the dark. In this regard, Pinto et al. evaluated the color of a cream containing *C. sativa* peel extract for 30 days. After 30 days of storage, the cream showed a mean variation of 3, 1.72, and 3.54 units for the parameters L*, a*, and b*, respectively. These variations were higher than those obtained in the present study after 30 days [95]. In addition, Balboa et al. formulated a cream with *Sargassum muticum* as a bioactive ingredient, and after evaluating its color, the L* parameter showed a reduction of between 7% and 31%, considerably higher than that obtained in our formulations (<5%) [105].

**Figure 9:**
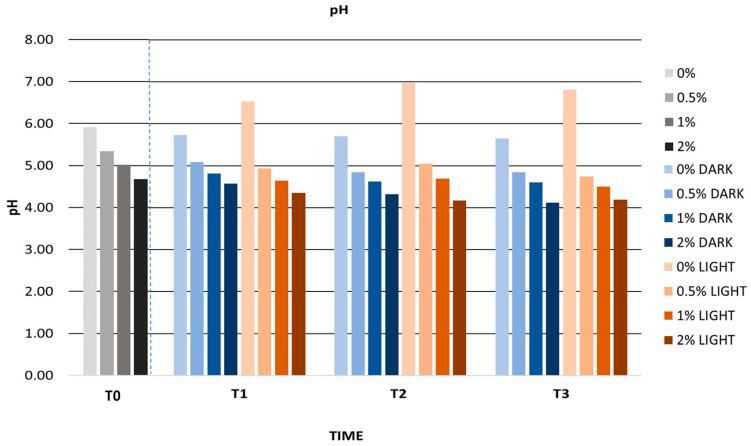
pH values during long-term stability. 0%: gel without extract; 0.5%: gel with 0.5% mango peel extract: 1%: gel with 1% mango peel extract; 2%: gel with 2% mango peel extract; DARK: samples stored in the dark; LIGHT: samples stored in the light. The dashed line indicates the separation between the control and the samples during long term stability.

Figure 9 shows the pH values during storage of the gels in the light and in the dark. The pH of the formulations remained constant over time with the exception of the pH of the gel without extract stored in the light, which increased from 5.92 to 6.98. In addition, all formulations showed a pH between 4 and 7, compatible with the skin. Previously, Hwang et al. formulated a lotion with *Prunus padus* bark extract as the active ingredient and reported pH changes after 28 days at 25 °C. The lotion showed a pH difference of 0.50 from an initial pH of 5.70 to a final pH of 5.42. These pH changes were similar to those obtained in the present study after 30 days [106]. Huma et al. studied the pH variation of a cream with beetroot extract for 60 days and observed a variation of 0.90, considering the formula stable. Taking into account that our results show a pH variation lower than that of Huma et al., we could consider the formulation developed in the present study as stable [107].

**Figure 10:**
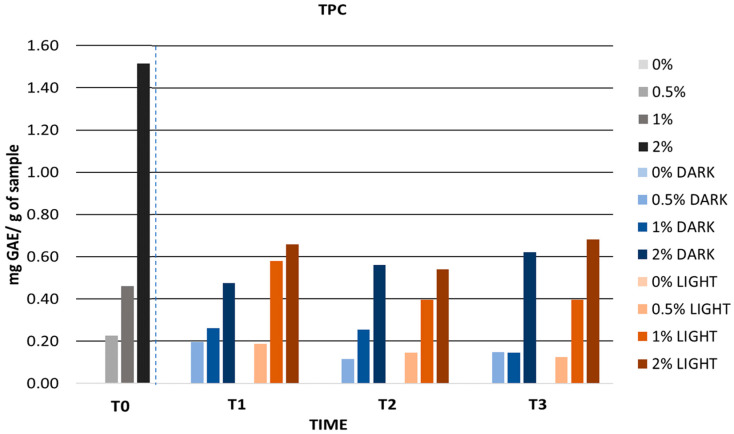
TPC values during long-term stability. 0%: gel without extract; 0.5%: gel with 0.5% mango peel extract: 1%: gel with 1% mango peel extract; 2%: gel with 2% mango peel extract; DARK: samples stored in the dark; LIGHT: samples stored in the light. The dashed line indicates the separation between the control and the samples during long term stability.

Figure 10 shows the TPC values of the gels stored in the light and in the dark. During the first month, the TPC values of the gels decreased, but from the second month onwards they remained constant. This indicates that the mango peel extract retained its bioactivity for 30 days and then decreased. This decrease could be related to the degradation of bioactive compounds by various factors such as storage time and temperature. Pinto et al. carried out the development of hydrogels containing *C. sativa* peel extract at different concentrations and observed how after 30 days the TPC values decreased with respect to the initial values (from 2.48 to 1.96 mg GAE/g sample) [108].

The long-term stability of the gels stored in light and dark was characterized by the absence of phase separation, homogeneous appearance of the gels, and pH values compatible with topical application. From the results obtained, it is possible to affirm that the gel formulations including mango peel extract at different concentrations showed good physical and bioactive stability.

## 4. Conclusions

This study evaluated the potential of mango peel as a source of skin-healthy bioactive compounds. HPLC-ESI-qTOF-MS analysis tentatively identified 71 compounds in the mango peel extract. Hydrolyzed tannins were the most representative group in mango peel. The mango peel extract showed remarkable antioxidant effects, highlighting the ability to donate electrons (FRAP) and transfer hydrogen atoms (ORAC) and the ability to inhibit radical species (HOCl and ^•^NO). Mango peel extract was able to effectively inhibit tyrosinase, elastase, collagenase, HYALase, and XO enzymes. In addition, the mango peel extract was incorporated into a cosmetic gel formulation with good physicochemical properties. The stability of the formulation was ensured by accelerated stability tests and over a time period of three months of storage in light and dark. The formulations were shown to be stable during accelerated stability and long-term stability. In addition, the TPC values of the gels containing 0.5% and 1% mango peel extract remained constant over time. However, this stability study was performed on a preliminary basis to obtain information on the developed formulation and its complexity. With this study, it will be possible to establish an adequate stability protocol considering all the factors that influence stability and through subsequent quality and stability tests. From a future perspective, it would be interesting to continue studying the benefits of mango peel extract on skin health and to continue advancing in the development of new cosmeceuticals through the incorporation of mango peel extract in cosmetic formulations and its subsequent in vivo study.

## Figures and Tables

**Table 1:** List of ingredients of the gel formulation.

Commercial Name	INCI Name	CAS Code	Function	%
Demineralized water	Water	7732-18-5	Aqueous phase	89.97, 89.47, 88.97, 87.97
Glycerine	Glycerine	56-81-5	Moisturizer/Emollient	5
Vegetal extract	-	-	Active ingredient	0, 0.5, 1, 2
Xanthan gum	Xanthan gum	11138-66-2	Emulsifier/Gelling agent	2
Ethanol	Alcohol	64-17-5	Solvent/Preservative	3
Citric acid	Citric acid	77-92-9/5949-29-1	Ph regulator	0.03

INCI: International nomenclature of cosmetic ingredients.

**Table 3:** Evaluation of total phenolic content, antioxidant capacity, free-radical scavenging, and enzymatic inhibition of mango peel extract.

Methodology	MP Extract	EPI	GA	KA	ELA	PHE
TPC (mg GAE/g DE)	42.20 ± 0.07	-	-	-	-	-
FRAP (mmol FeSO_4_/g DE)	0.659 ± 0.004	-	-	-	-	-
TEAC (μmol TE/g DE)	484.3 ± 0.7	-	-	-	-	-
ORAC (mmol TE/g DE)	0.393 ± 0.008	-	-	-	-	-
O_2_^•^^−^ (mg/L) ^1^	950 ± 10	70 ± 5	50 ± 3	-	-	-
^•^NO (mg/L) ^1^	7.5 ± 0.3	0.87 ± 0.02	1.4 ± 0.3	-	-	-
HOCl (mg/L) ^1^	50 ± 10	0.18 ± 0.01	3.8 ± 0.3	-	-	-
Tyrosinase	170 ± 16 ^1^	-	-	49 ± 6 ^2^	-	-
Elastase	880 ± 10 ^1^	-	-	-	53 ± 5 ^3^	-
HYALase (mg/L)	27 ± 2 ^1^	167 ± 6 ^4^	102 ± 4 ^4^	-	-	-
Collagenase (mg/L) ^1^	260 ± 20	-	-	-	-	83 ± 2
XOD (mg/L) ^1^	2 ± 1	9 ± 1	-	-	-	-

MP: mango peel; FRAP: ferric reducing antioxidant power assay; TEAC: Trolox equivalent antioxidant capacity; ORAC: oxygen radical absorbance capacity; GAE: gallic acid equivalent; DE: dry extract; TE: Trolox equivalent. Data are means ± standard deviation (*n* = 3). HYALase: hyaluronidase; EPI: epicatechin; GA: gallic acid; KA: Kojic acid; ELA: elastatinal; PHE: 1, 10-phenanthroline; inh.: inhibition. ^1^ Inhibitory Concentration at 50% (mg/L). ^2^ % Inhibition at 21.3 mg/L. ^3^ % Inhibition at 51.26 mg/L. ^4^ Inhibitory Concentration at 20% (mg/L).

**Table 4:** Initial values of the physicochemical parameters of the gels.

Parameters	0%	0.5%	1%	2%
**Viscosity (Pa·s)**				
10 rpm	8.4 ± 0	9.87 ± 0.12	11.1 ± 0	13.63 ± 0.06
70 rpm	1.67 ± 0.01	1.87 ± 0.02	2.06 ± 0.02	2.56 ± 0.01
**Color**				
L*	25.73 ± 0.02	24.65 ± 0.02	23.30 ± 0.02	23.00 ± 0.02
a*	−0.58 ± 0.01	−1.11 ± 0.04	−0.62 ± 0.02	−0.4 ± 0.03
b*	0.88 ± 0.06	5.11 ± 0.03	5.38 ± 0.04	5.32 ± 0.02
**pH**	5.92 ± 0.01	5.34 ± 0.02	5.01 ± 0.05	4.67 ± 0.02
**TPC (mg GA/g DE)**	0	0.23 ± 0.05	0.46 ± 0.05	1.51 ± 0.07

Data are means ± standard deviation (*n* = 3). Rpm: revolutions per minute; GAE: gallic acid equivalent; DE: dry extract; 0%: gel without extract; 0.5%: gel with 0.5% mango peel extract: 1%: gel with 1% mango peel extract; 2%: gel with 2% mango peel extract.

## Data Availability

The data presented in this study are available on request from the corresponding author.
